# Association Between T_2_^*^ Relaxation Times Derived From Ultrashort Echo Time MRI and Symptoms During Exercise Therapy for Patellar Tendinopathy: A Large Prospective Study

**DOI:** 10.1002/jmri.27751

**Published:** 2021-05-30

**Authors:** Stephan J. Breda, Robert‐Jan de Vos, Dirk H. J. Poot, Gabriel P. Krestin, Juan A. Hernandez‐Tamames, Edwin H. G. Oei

**Affiliations:** ^1^ Department of Radiology & Nuclear Medicine Erasmus MC University Medical Center Rotterdam The Netherlands; ^2^ Department of Orthopedics & Sports Medicine Erasmus MC University Medical Center Rotterdam The Netherlands

**Keywords:** quantitative MRI, T_2_^*^ relaxometry, patellar ligament, tendons, athletes, exercise therapy

## Abstract

**Background:**

Exercise therapy is considered preferential treatment for patellar tendinopathy (PT). However, there is conflicting evidence for structural patellar tendon adaptation in response to exercise therapy and its association with symptoms is weak.

**Purpose:**

To assess the association between 1) T_2_^*^ relaxation times and symptom severity; 2) baseline T_2_^*^ and clinical outcome; and 3) longitudinal T_2_^*^ changes and clinical outcome in athletes with PT performing exercise therapy.

**Study Type:**

Randomized controlled clinical trial.

**Subjects:**

Seventy‐six athletes (18–35 years) with clinically diagnosed and ultrasound‐confirmed PT.

**Field strength/Sequence:**

3D gradient echo sequence (3.0 T).

**Assessment:**

Patients were enrolled in a randomized trial of progressive tendon‐loading exercises (PTLE) versus eccentric exercise therapy (EET). Symptoms were assessed using the Victorian Institute of Sports Assessment (VISA‐P) questionnaire. 3D‐Ultrashort echo time (UTE)‐MRI was acquired at baseline, 12 and 24 weeks. Voxel‐wise T_2_^*^ relaxation times were quantified using mono‐exponential and bi‐exponential models. T_2_^*^ analysis was performed in three patellar tendon tissue compartments representing: aligned collagen, degenerative tissue, and interface.

**Statistical Tests:**

Adjusted general linear, mixed‐linear models, and generalized estimating equations.

**Results:**

We included 76 patients with PT (58 men, mean age 24 ± 4 years); 38 in the PTLE‐group and 38 in the EET‐group, of which 57 subjects remained eligible for analysis. T_2_^*^ relaxation times were significantly associated with VISA‐P in degenerative and interface tissues of the patellar tendon. No association was found between baseline T_2_^*^ and VISA‐P after 24 weeks (*P* > 0.29). The estimated mean T_2_^*^ in degenerative tissue decreased from 14 msec (95%CI: 12–16) at baseline to 13 msec (95%CI: 11–15) at 12 weeks and to 13 msec (95%CI: 10–15) at 24 weeks. The significant decrease in T_2_^*^ from baseline to 24 weeks was associated with improved clinical outcome.

**Data Conclusion:**

Tissue‐specific T_2_^*^ relaxation times, identified with 3D‐UTE‐MRI, decreased significantly in athletes with patellar tendinopathy performing exercise therapy and this decrease was associated with improved clinical outcome.

**Evidence Level:**

1

**Technical Efficacy:**

Stage 4

## Introduction

Patellar tendinopathy (PT) is a common disorder due to tendon overuse injury in athletes.^1^ PT is diagnosed, based on localized pain at the attachment of the patellar tendon to the patellar bone.^2^ Symptoms occur with tendon loading, such as jumping, landing, and cutting in sports activities and activities of daily living, or physically demanding work.^3^, ^4^ Symptoms from PT can be assessed using the validated Victorian Institute of Sports Assessment (VISA‐P) questionnaire, with an outcome ranging from 0 to 100 points. A score of 100 indicates no pain, maximum function and unrestricted ability to play sports.^5^ Conservative treatment of PT consists of exercise therapy and is focused on increasing the tendon's capacity to tolerate load.^6^

The pathophysiology of PT is largely unknown but involves characteristic degenerative changes at the latter stages.^7^ The hierarchical architecture of the patellar tendon normally consists of parallel ordered collagen fibers and ground substance.^8^ Proteoglycans are composed of multiple glycosaminoglycan chains that are attached to a protein core and are found in the extracellular matrix of tendons.^9^ Highly negatively charged proteoglycans attract water and contribute to compressive resistance within the tissue.^10^ In tendinopathy, there is an increase in cellularity and ground substance volume.^11^ Exercise therapy is assumed to reverse this degenerative cascade.^12^ However, there is conflicting evidence that exercise therapy results in structural adaptation measured with clinically available imaging modalities (eg, ultrasound and MRI).^13^ Although the correlation between imaging outcomes and clinical outcomes remains unclear, the glycosaminoglycan content from tendon biopsies correlates well with tendon pain.^13^, ^14^

Imaging of tendons using MRI with conventional pulse sequences is typically limited by the fast free induction decay of collagen, commonly only visualizing increased signal intensity in the proximal patellar tendon, which may reflect increased water content.^15^ Strong spin–spin interactions usually lead to undetectable signal from short T_2_^*^ relaxation components, such as collagen.^16^

Ultrashort echo time (UTE) MRI facilitates the detection of signal from short T_2_^*^ tissues such as tendons, which can be used to infer tendon hydration state by voxel‐wise T_2_^*^ quantification.^17^ T_2_^*^ analysis reflects signal from protons in different water pools within the patellar tendon, consisting of collagen‐bound water pools (shorter T_2_^*^ relaxation times) and free water pools (longer T_2_^*^ relaxation times).^18^ While mono‐exponential T_2_^*^ fitting models have shown the best reliability, bi‐exponential models allow differentiating voxels in highly organized collagen compartments from degenerative tissue compartments within the patellar tendon.^19^, ^20^

Previous studies on UTE in tendinopathy have consistently shown increased T_2_^*^ relaxation times.^21^, ^22^ However, quantification of T_2_^*^ changes in PT within specific tissue compartments has not been performed. Moreover, associations with symptoms have not been studied. We hypothesized that temporal changes in T_2_^*^ can be detected using UTE‐MRI and might be associated with clinical outcome in athletes with patellar tendinopathy performing exercise therapy.

The first aim of this study was to investigate the association between T_2_^*^ relaxation times within different tissue compartments of the patellar tendon and symptom severity. The second aim was to investigate the association between baseline T_2_^*^ and clinical outcome after exercise therapy. The third aim was to evaluate the association between longitudinal T_2_^*^ changes and changes in severity of symptoms in athletes with PT.

## Materials and Methods

### Study Participants

Ethical approval was obtained by the institutional review board and all participants provided written informed consent. Participants enrolled in the JUMPER‐study, a randomized controlled trial investigating the effect of progressive tendon‐loading exercises (PTLE) vs. eccentric exercise therapy (EET) for PT (ClinicalTrials.gov ID: NCT02938143). Inclusion criteria were age 18–35 years; history of knee pain in the patellar tendon region associated with training and competition; playing sports ≥3 times a week before injury onset; tenderness on palpation of the proximal patellar tendon; structural tendon changes on grayscale ultrasound and/or increased tendon vascularity on power Doppler; Victorian Institute of Sports Assessment (VISA‐P) score < 80/100. Exclusion criteria are presented in the trial register. Activity level was measured using the Cincinnati Sports Activity Scale (CSAS).^23^

### Inclusion Protocol

After an initial screening of online applications from study advertisements, potentially eligible athletes were invited to our hospital for medical history taking and physical examination performed by one sports physician (R.V.) with 10 years' experience. Subsequently, grayscale ultrasound (including anteroposterior tendon thickness and the presence of hypoechoic regions, tendon calcifications and erosions of the inferior patellar border) and power Doppler ultrasound (PDUS) were performed by the main investigator, a radiologist‐in‐training with 5 years' experience (S.B.) under supervision of a senior musculoskeletal radiologist with 16 years' experience (E.O.) to confirm the clinical diagnosis PT. Ultrasound was regarded conclusive for PT when structural changes to normal parallel ordered collagen fibers and/or hypoechoic changes and/or tendon thickening (anterior–posterior diameter > 6 mm) were confirmed and/or presence of intratendinous Doppler flow was detected on PDUS.^24^

### Interventions

The intervention group performed progressive tendon‐loading exercises (PTLE) within limits of acceptable pain, consisting of four consecutive stages (isometric, isotonic, plyometric, and sport‐specific exercises).^3^ The control group performed usual care eccentric exercise therapy (EET), which typically provokes substantial pain.^25^ Centralized computer‐based randomization was performed in a 1:1 ratio to PTLE or EET, using computer‐generated block randomization with a variable block size ranging from 4 to 10. Patients in both study arms were also instructed to perform exercises targeting risk factors for PT. All patients received detailed advice and education on tendon care. Modification of pain‐provoking athletic activities was advised for at least 4 weeks and we advised to perform (sports) activities within the limits of acceptable pain (pain score ≤ 3 points on a scale 0–10). Details regarding these therapeutic interventions are published elsewhere.^26^ The main investigator (S.B.) was blinded for the allocated treatment during the entire period of data collection.

### Outcomes

Clinical and imaging outcomes were collected at baseline, 12 and 24 weeks by the main investigator (S.B.). Patients were included in the primary analysis when there was at least one follow‐up measurement available.

### Clinical Outcome

Symptom severity was assessed using the validated Victorian Institute of Sports Assessment (VISA‐P) questionnaire prior to image acquisition at every visit.^5^ The main investigator was blinded for VISA‐P scores at the time of image acquisition and analysis.

### Image Acquisition

Imaging was performed at 3.0 T (Discovery MR750, GE Healthcare, Waukesha, WI, USA) using a 16‐channel flexible coil (NeoCoil, Pewaukee, WI, USA) and a fixation device. The knee was positioned in 30° flexion, using a cylindrical tube and foam padding.^19^

Using a research prototype 3D‐UTE‐Cones sequence (GE Healthcare), 16 echoes (0.032, 0.49, 0.97, 2.92, 4.87, 6.82, 8.77, 10.72, 12.67, 14.62, 16.57, 18.52, 20.47, 22.42, 24.37, 26.32 msec) were acquired using four multi‐echo sequences with a constant repetition time of 83.4 msec.^27^ The axial 3D‐UTE‐Cones sequence parameters were acquisition time = 13:15 minutes per multiecho sequence, field‐of‐view (FOV) = 15 cm, matrix size = 252 × 252, voxel size 0.6 × 0.6 × 1.5 mm^3^, number of slices = 60, number of excitations (NEX) = 1, bandwidth 125 kHz, flip angle = 17°, and two excitations per fat saturation. Echoes were scanned in interleaved order using four fat saturated multiecho UTE‐acquisitions with a total scan time of 53 minutes. The full acquisition protocol is described elsewhere.^19^ This final protocol was implemented after changing our previously designed image protocol that consisted of seven single echo acquisitions and one multiecho acquisition in the coronal oblique plane without fat saturation (data not included), in order to increase SNR for T_2_^*^ quantification.

### Image Preparation

Image registration was performed to facilitate spatial one‐to‐one mapping of voxels across longitudinal UTE‐acquisitions, using in‐house developed tools (Elastix v.4.8, Rotterdam, The Netherlands) and Matlab software (R2015b; TheMathWorks, Natick, MA, USA).^28^ First, rigid registration was performed on the entire knee volume, which corrected for rotation and translation between multiecho acquisitions and examinations from baseline and follow‐up visits. Second, groupwise nonlinear refinement registration was performed on a volume of interest drawn on three orthogonal views, including only the anterior knee part including the patellar tendon.^29^

### Image Analysis

Image analysis was performed by the main investigator, a radiologist‐in‐training with 5 years' experience (S.B.) under supervision of a postdoctoral researcher specialized in image analysis with 14 years' experience (D.P.) using an in‐house developed Matlab script. For quantitative T_2_^*^ analysis, mono‐exponential and bi‐exponential models were fitted to registered UTE‐images, using maximum likelihood estimation incorporating the Rician noise model.^20^, ^30^ The initial step after fitting the data was to manually segment the outer margins of the patellar tendon on 10 consecutive slices covering the proximal patellar tendon, as described elsewhere.^19^

Second, the T_2_^*^ data within this mask were categorized into three groups, by selecting voxels based on the average over visits of the percentage of short T_2_^*^ components from the bi‐exponential model (0%–30%, 30%–60%, and 60%–100% short T_2_^*^).^19^ These three subregions that together spanned the manually drawn mask represented: 1) mostly short T_2_^*^ (60%–100% short), 2) mostly long T_2_^*^ (0%–30% short), and 3) an interface that separated the two (30%–60% short). The subregions were considered to represent aligned collagen, degenerative tissue, and an interface, respectively and were based on histogram analysis of the T_2_^*^ frequency distribution in previous work.^19^ Finally, the corresponding mono‐exponential T_2_^*^ relaxation times were used, in these selected voxel groups that were constant over visits, to calculate T_2_^*^ relaxation times for each voxel group (patellar tendon subregion) accordingly. Thus, the selected voxel groups that delineated the regions of interest used for analysis were defined on the baseline scans and propagated to follow‐up scans to assess temporal changes in T_2_^*^ on subsequent scans (after 12 weeks and 24 weeks). The mono‐exponential T_2_^*^ relaxation times were fitted voxel‐wisely in the entire registered scan volume from three separate visits.

### Statistical Analysis

Normality of data was assessed using the Shapiro–Wilk test and homogeneity of variances was tested using the Levene test. Associations between T_2_^*^ relaxation times and VISA‐P score were assessed using multiple linear regression analyses. Adjusted general linear models were used to assess associations between baseline T_2_^*^ and clinical outcome after 24 weeks. Longitudinal data were analyzed using adjusted generalized estimating equations (GEE) models, to estimate population‐averaged effects. Whole group analyses were performed and between‐group differences in relation to the time course of the dependent variables were evaluated using an interaction term “study arm^*^visit” in the GEE model, where the visit defined baseline, 12 weeks or 24 weeks. Bonferroni corrections were applied in the GEE‐models for the following comparisons: 1) baseline vs. 12 weeks, 2) 12 weeks vs. 24 weeks, and 3) baseline vs. 24 weeks. Associations with clinical outcome were evaluated using adjusted mixed linear models. All models were adjusted for predefined potential confounding factors, including age, sex, BMI, CSAS, and symptom duration. All analyses were performed following an intention‐to‐treat principle. Imputation of missing data was not performed. Instead, posthoc sensitivity analyses using the last observation carried forward (LOCF) approach were performed when the amount of missing data exceeded 5% of the total number of observations.^31^ Statistical analysis was performed using IBM SPSS software version 25 (IBM Corp., Armonk, NY, USA). Statistical significance was defined as a *P*‐value < 0.05.

## Results

### Study Population

**FIGURE 1:**
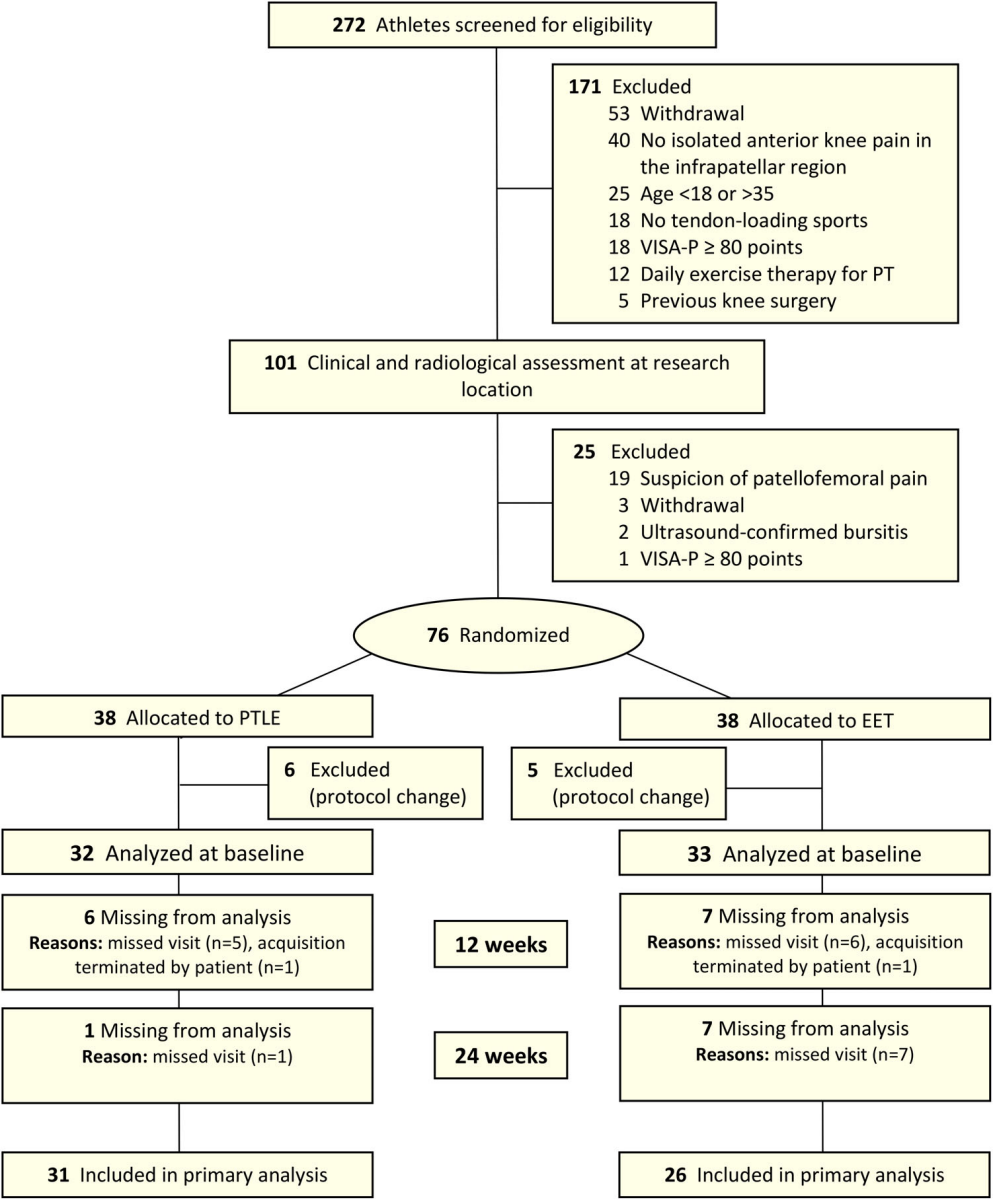
The consort flow diagram. PT = patellar tendinopathy; VISA‐P = Victorian Institute of Sports Assessment questionnaire for patellar tendons.

Athletes were consecutively enrolled between January 2017 and June 2019. A total of 76 included athletes (58 men, mean age 24 ± 4 years) with clinically diagnosed and ultrasound‐confirmed PT were included of which 38 were randomized to PTLE and 38 to EET (Fig. 1). Due to a change in the MR acquisition protocol during the study period, 11 subjects were missing for T_2_^*^ analysis. Furthermore, eight subjects could not be included in the primary analysis due to missing data. Demographic characteristics of the study population are listed in Table 1.

**TABLE 1:** Baseline Characteristics

Characteristics	Whole group (*n* = 76)	PTLE Group (*n* = 38)	EET Group (*n* = 38)
Age, mean (SD), years	24 (3.8)	24 (3.5)	24 (4.2)
Sex, male	58 (76)	31 (82)	27 (71)
BMI, mean (SD)	23.9 (2.9)	23.8 (2.5)	24.1 (3.2)
Symptom duration, median [IQR], weeks	104 [43–208]	119 [64–273]	78 [40–169]
VISA‐P score, mean (SD)	55 (13.1)	55 (13.1)	56 (13.2)
Sports Activity Scale (CSAS) prior to onset of PT			
Level I (4 days/week–7 days/week)			
100	17 (22)	10 (26)	7 (18)
95	0 (0)	0 (0)	0 (0)
90	0 (0)	0 (0)	0 (0)
Level II (1days/week–3 days/week)			
85	50 (66)	23 (61)	27 (71)
80	9 (12)	5 (13)	4 (11)
Sports participation in desired sport at the time of study commencement, *n* (%)			
Equal	19 (25)	10 (26)	9 (24)
Reduced	29 (38)	14 (37)	15 (40)
Ceased	28 (37)	14 (37)	14 (37)
Affected side			
Unilateral, left/right, *n* (%)	26 (34) / 18 (24)	10 (26) / 9 (24)	16 (42) / 9 (24)
Bilateral, *n* (%)	32 (42)	19 (50)	13 (34)
US‐assessment			
AP thickness, mm ± SD	8.4 ± 2.3	8.2 ± 2.7	8.6 ± 2.0
Hypoechoic regions, *n* (%)	76 (100)	38 (100)	38 (100)
Tendon calcifications, *n* (%)	20 (26)	9 (24)	11 (29)
Patellar erosions, *n* (%)	24 (32)	17 (45)	7 (18)
Power Doppler			
0: absence of Doppler flow	7 (9)	5 (13)	2 (5)
1: Doppler flow posterior to tendon	0 (0)	0 (0)	0 (0)
2: 1–2 intratendinous blood vessels	18 (24)	12 (32)	6 (16)
3: 3–4 intratendinous blood vessels	7 (9)	3 (8)	4 (11)
4: network of blood vessels	44 (58)	18 (47)	4 (68)

PTLE = progressive tendon‐loading exercise therapy; EET = heavy‐load eccentric exercise therapy; SD = standard deviation; BMI = body mass index; IQR = interquartile range; VISA‐P = Victorian Institute of Sports Assessment Questionnaire for patellar tendons; CSAS = Cincinnati Sports Activity Scale; PT = patellar tendinopathy; n = number; US = ultrasound; AP = anterior–posterior.

Data are presented as No. (%) unless otherwise specified.

### Clinical Outcome

Among all athletes, the estimated mean VISA‐P score improved significantly from 57 (95% CI: 53–61) at baseline to 72 (95% CI: 67–76) at 12 weeks and 80 (95% CI: 76–84) at 24 weeks follow‐up. In the PTLE group, the estimated mean VISA‐P score improved significantly from 56 (95% CI: 52–61) at baseline to 84 (95% CI: 79–89) at 24 weeks and in the EET‐group it improved significantly from 57 (95% CI: 53–62) to 75 (95% CI: 69–82). The parameter estimate for the “study arm^*^visit” interaction using GEE was statistically significant. The homogeneity of variance assumption was not violated (*P* = 0.59). The significant adjusted mean between‐group difference of the VISA‐P score at 24 weeks was 9 points (95% CI: 1–16), in favor of the PTLE group, indicating an improved clinical outcome in athletes performing progressive tendon‐loading exercises (PTLE).^26^

### Association between T_2_^*^ in Different Tissue Compartments and Symptom Severity

**FIGURE 2:**
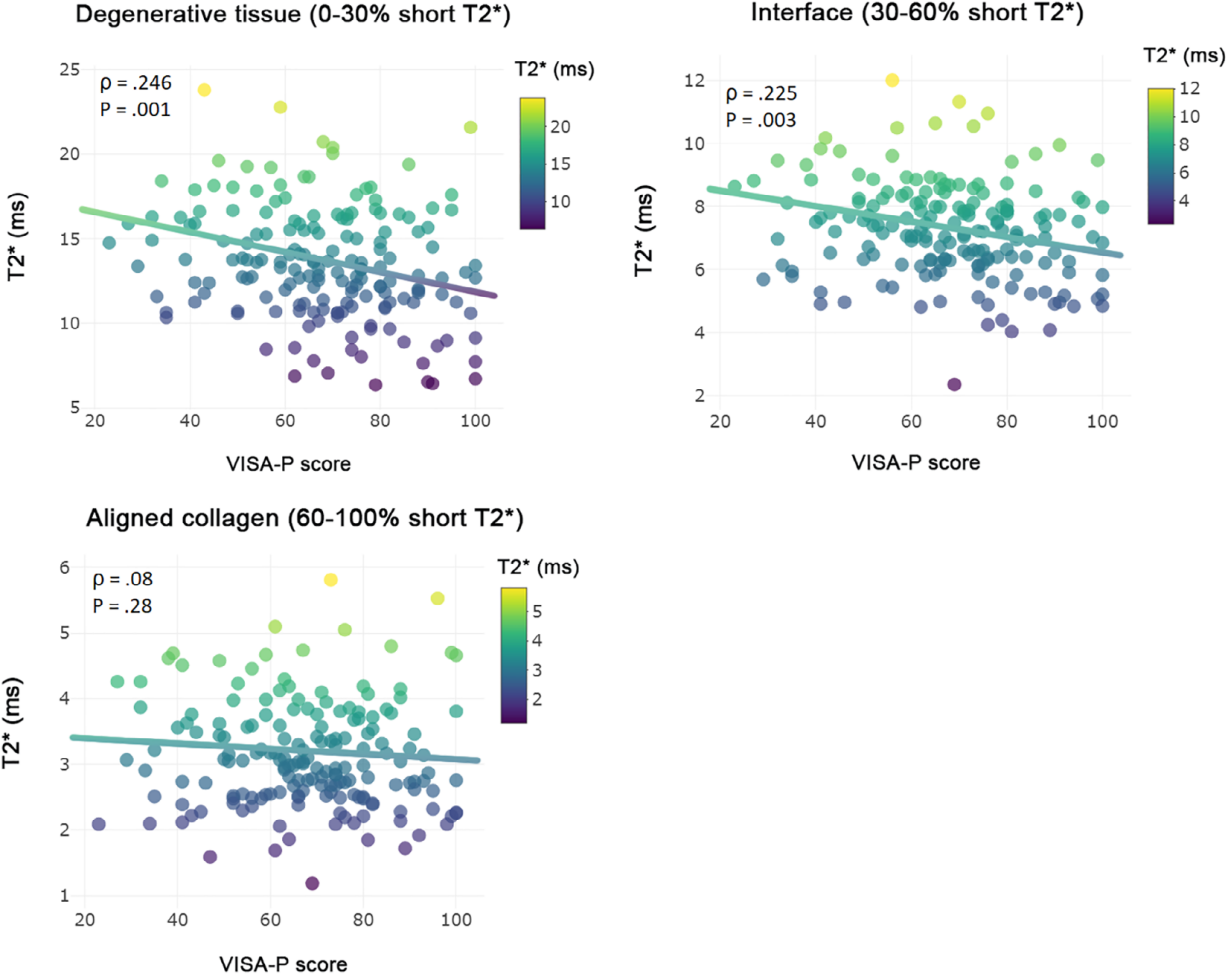
Association between mono‐exponential T_2_^*^ in specific tissue compartments and VISA‐P score. Scatter plot of individual T_2_^*^ relaxation times versus symptom severity as scored at baseline and after 12 and 24 weeks with the validated VISA‐P questionnaire for patellar tendinopathy. Plots illustrate a significant negative association between mono‐exponential T_2_^*^ and VISA‐P score in degenerative tissue and interface of the patellar tendon, but not in aligned collagen.

Adjusted linear regression analysis demonstrated a statistically significant linear association between VISA‐P score and T_2_^*^ relaxation times in both degenerative tissue and in the interface between aligned collagen and degenerative tissue (Table 2). Scatter plots of individual associations between mono‐exponential T_2_^*^ in specific tissue‐compartments and VISA‐P score are illustrated in Fig. 2.

**TABLE 2:** Association Between VISA‐P and T_2_^*^ (Multiple Linear Regression Analysis)

	Degenerative tissue (0%–30% short T_2_^*^)			Interface (30%–60% short T_2_^*^)			Aligned collagen (60%–100% short T_2_^*^)		
Factor	β	95% CI	*P* value	β	95% CI	*P* value	β	95% CI	*P* value
Age	0.008	−0.125 to 0.141	0.908	0.023	−0.040 to 0.085	0.472	0.043	0.011–0.076	<0.05
Sex	1.460	0.300–2.620	<0.05	0.422	−0.121 to 0.965	0.127	0.049	−0.234 to 0.332	0.732
BMI	0.015	−0.175 to 0.205	0.876	0.026	−0.063 to 0.115	0.561	0.052	0.006–0.098	<0.05
Duration	<0.001	−0.004 to 0.004	0.989	0.001	−0.001 to 0.002	0.482	0.001	0.000–0.002	<0.05
CSAS	−0.044	−0.119 to 0.031	0.252	0.010	−0.025 to −0.006	0.565	0.006	−0.012 to 0.025	0.506
VISA‐P	**−0.046**	**−0.075 to −0.018**	**<0.05**	**−0.019**	**−0.033 to −0.006**	**<0.05**	−0.003	−0.010 to 0.004	0.461

CI = confidence interval; BMI = body mass index; CSAS = Cincinnati Sports Activity Scale; VISA‐P = Victorian Institute of Sports Assessment Questionnaire for patellar tendons.

Adjusted mean differences that were significant at 0.05 level after Bonferroni correction are in bold.

### Association between Baseline T_2_^*^ and Change in Symptom Severity

Baseline T_2_^*^ values for all tissue compartments are listed in Table 3. There was no significant association of baseline T_2_^*^ with clinical outcome after 24 weeks of exercise therapy for all of the tissue compartments of the patellar tendon (degenerative tissue [*R* ^2^ = 0.17, *P* = 0.29], aligned collagen [*R* ^2^ = 0.17, *P* = 0.95], and the interface compartment [*R* ^2^ = 0.15, *P* = 0.55]).

**TABLE 3:** Unadjusted and Adjusted Change in T_2_^*^ Over Time

	Unadjusted T_2_^*^ (msec) ± SD (raw data)				Adjusted Mean Difference (95% CI), From Baseline^a^	
T_2_^*^ subregion	Baseline	12 weeks	24 weeks		12 weeks	24 weeks
Degenerative tissue (0%–30% short T_2_^*^)	14.2 ± 3.2	13.5 ± 3.4	12.8 ± 3.5		−0.7 (−1.3 to −0.1)	**−1.3 (−2.0 to −0.6)**
Interface (30%–60% T_2_^*^)	7.5 ± 1.6	7.3 ± 1.5	6.9 ± 1.5		−0.3 (−0.7 to 0.2)	−0.6 (−1.1 to −0.1)
Aligned collagen (60%–100% T_2_^*^)	3.1 ± 0.9	3.2 ± 0.6	3.1 ± 0.9		0.1 (−0.2 to 0.3)	−0.0 (−0.2 to 0.2)

^a^ The adjusted mean differences were calculated using generalized estimating equations (GEE) with adjustments for the following predefined baseline variables: age, sex, BMI, symptom duration and Cincinnati Sports Activity Scale.

Adjusted mean differences that were significant at 0.05 level after Bonferroni correction in bold.

### Longitudinal UTE‐MRI Data and Relation with Clinical Outcome

**FIGURE 3:**
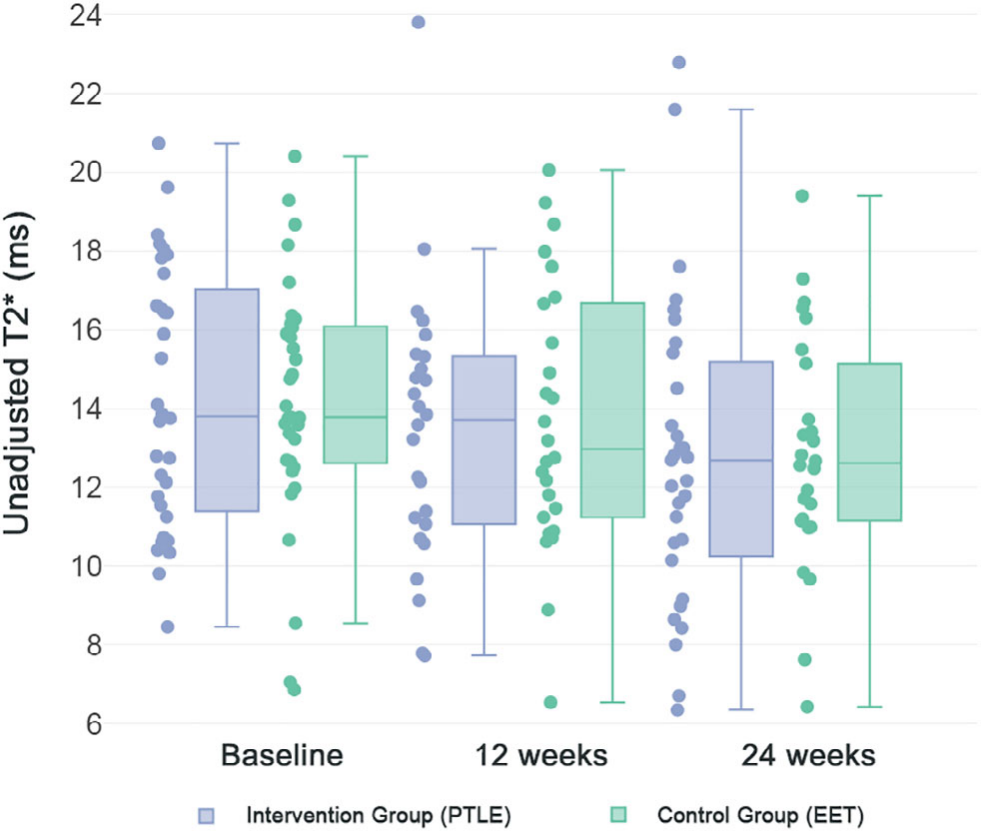
Longitudinal T_2_^*^ changes in degeneratie tissue. Box plot and scatter diagram of longitudinal T_2_^*^ measurements in the degenerative tissue of the patellar tendon. Results are divided by study arm (progressive tendon‐loading exercises [PTLE] and eccentric exercise therapy [EET]). The midline of the boxplot represents the median of the data, with the upper and lower limits of the box being the 25th and 75th percentile. The whiskers extend up to 1.5 times the interquartile range.

**FIGURE 4:**
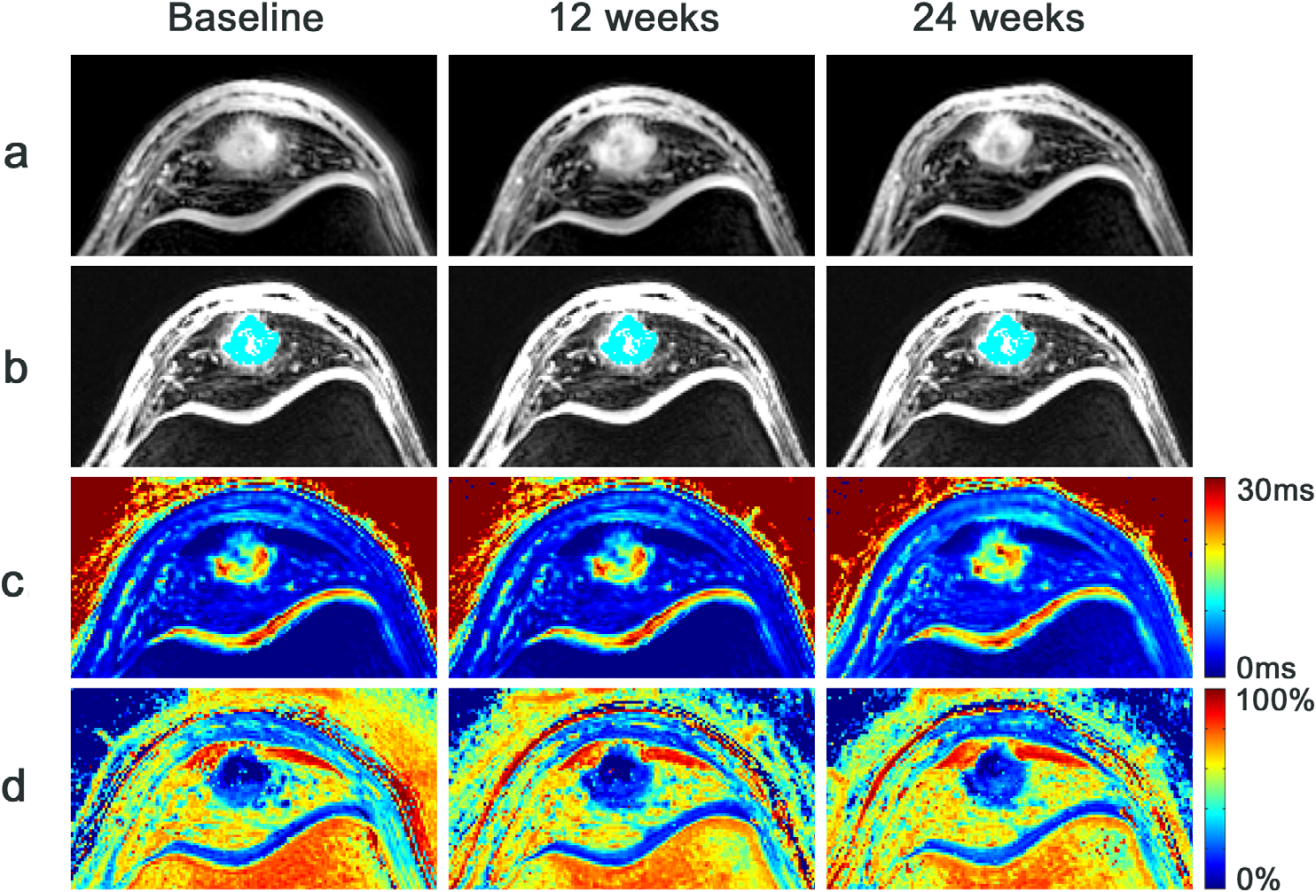
Illustration of longitudinal T_2_^*^ analysis. (a) Transverse 3D‐UTE images (TE = 4.82 msec) of the proximal patellar tendon in a 19‐year‐old male volleyball player with clinically diagnosed and ultrasound‐confirmed patellar tendinopathy. (b) Automatically selected voxels (cyan colored) for tissue‐specific T_2_^*^ analysis in the degenerative tissue of the patellar tendon, based on the bi‐exponential fitting threshold indicating 0%–30% short T_2_^*^ components. In this patient, mean T_2_^*^ decreased from 19.9 ± 7.3 msec (baseline) to 17.2 ± 5.8 msec (12 weeks) to 16.8 ± 4.9 msec (24 weeks). (c) Mono‐exponential T_2_^*^ maps, on a scale from dark blue (short T_2_^*^ relaxation times) to red (long T_2_^*^ relaxation times). (d) Bi‐exponential fitting maps, displaying the percentage of short T_2_^*^ components on a scale from dark blue (0% short T_2_^*^ components) to red (100% short T_2_^*^ components).

Among all athletes, a significant decrease in T_2_^*^ was found in the voxels that represented the degenerative tissue of the patellar tendon, from 14 ± 3 msec at baseline to 13 ± 4 msec at 24 weeks (adjusted mean difference [95% CI] = 1 msec [1–2]). The change in T_2_^*^ was not significant at 12 weeks (adjusted mean difference [95% CI] = 1 msec [0–1], *P* = 0.09). For voxels that represented aligned collagen and voxels that represented the interface between aligned collagen and degenerative tissue, the adjusted mean differences were not statistically significant (Table 3). The interaction term “study arm^*^visit” was not statistically significant for any of the tissue compartments (aligned collagen, *P* = 0.42; interface, *P* = 0.49; degenerative tissue, *P* = 0.54) (Fig. 3). The homogeneity of variance assumption was not violated for all tissue compartments (aligned collagen, *P* = 0.59; interface, *P* = 0.17; degenerative tissue *P* = 0.23). An example of the longitudinal T_2_^*^ change is illustrated in Fig. 4.

**FIGURE 5:**
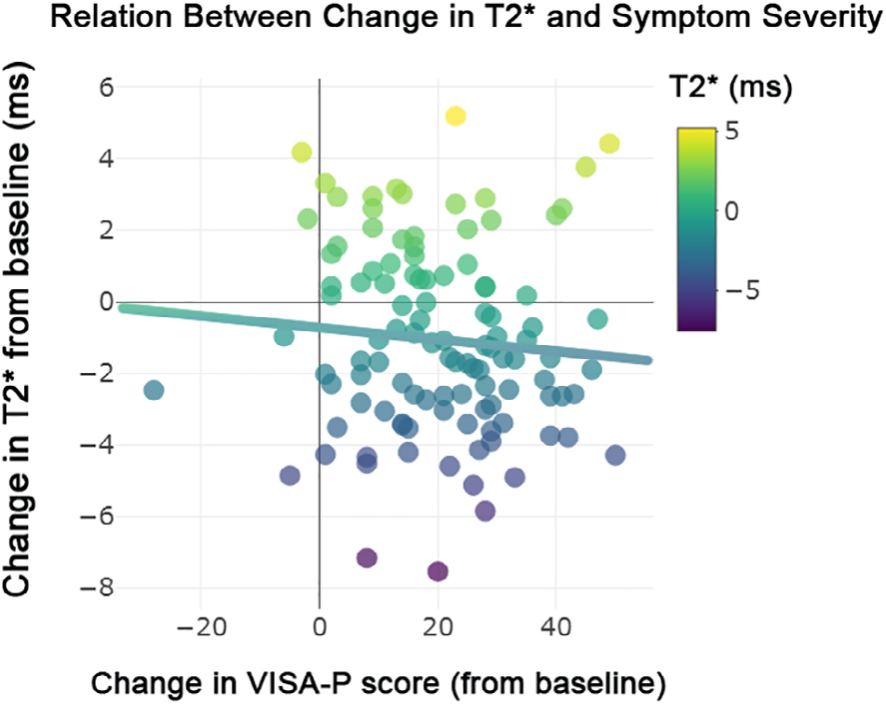
Relation between change in T_2_^*^ and symptom severity. There was a significant association between a decrease in mono‐exponential T_2_^*^ within the degenerative tissue and clinical improvement (*P* = 0.005). There was no association with clinical outcome for the aligned collagen (*P* = 0.77) and interface (*P* = 0.06) tissue compartments.

The significant T_2_^*^ decrease in the degenerative tissue compartment was significantly associated with the improvement in severity of symptoms as measured with the VISA‐P score (main effect, −1.2 [95% CI: −2.0 to −0.4]), Fig. 5. There was no association with clinical outcome for the aligned collagen (*P* = 0.77) and interface (*P* = 0.06) tissue compartments.

### Post hoc Sensitivity Analysis of Missing Data

Twenty one of the 195 UTE‐MRI acquisitions (11%) were missing (Fig. 1). Sensitivity analyses to assess the influence of missing data showed that the estimated mean VISA‐P score improved significantly from 56 points (95% CI: 53–60) at baseline to 69 points (95% CI: 65–73) at 12 weeks and 78 points (95% CI: 74–82) at 24 weeks follow‐up among all athletes. After 24 weeks, a significant adjusted mean between‐group difference of 11 points (95% CI: 3–18) was found. The T_2_^*^ decrease in degenerative tissue between baseline and 24 weeks follow up remained significant (mean difference, 1 [95% CI: 1–2]). The association with clinical outcome was significant (main effect, −1.1 [95% CI: −1.9 to −0.3]) when the most recent preceding T_2_^*^ value was carried forward to substitute all missing values. There were no significant T_2_^*^ changes in voxels that represented aligned collagen (main effect, 0.7 [95% CI: −2.1 to 3.4]; *P* = 0.64) and the voxels that represented the interface (main effect, −1.3 [95% CI: −2.7 to 0.1]; *P* = 0.07) between aligned collagen and degenerative tissue.

## Discussion

We found that T_2_^*^ relaxation times within the degenerative tissue of the patellar tendon were associated with symptom severity in athletes with patellar tendinopathy. There was no predictive value of T_2_^*^ at baseline for the clinical outcome after 24 weeks. Decreasing T_2_^*^ relaxation times were related to a better clinical outcome after 24 weeks using tissue‐specific T_2_^*^ analyses.

This randomized controlled trial demonstrated longitudinal changes in T_2_^*^ in patellar tendinopathy using a tissue‐specific analysis method based on bi‐exponential fitting parameters.^19^ The capability of UTE‐MRI to detect longitudinal changes had already been demonstrated by others, specifically in healing tendon grafts following anterior cruciate ligament reconstruction, in human cadaveric tendons that were subjected to tensile static loading, and in Achilles tendons of long distance runners.^32^, ^33^, ^34^

The small, but significant, decrease in T_2_^*^ relaxation times within the degenerative tissue may demonstrate the patellar tendon's ability to change structurally in response to exercise therapy. Conservative treatment options focus on structural adaptation of the patellar tendon to progressive loading of the tendon.^35^ However, the current evidence is conflicting regarding the effect of exercise therapy on structural adaptation measured with clinically available imaging modalities.^6^, ^13^ The significant association between decreased T_2_^*^ relaxation times and improved clinical outcome in our study further emphasizes the benefit of strengthening exercises in the treatment of tendinopathy.^3^ The hydration state of the degenerative tissue as quantified with T_2_^*^ mapping that was significantly associated with symptom severity was in accordance with a previous study that found an association of glycosaminoglycan content from tendon biopsies with VISA‐P score.^14^ Subregional T_2_^*^ quantification, facilitating the monitoring of tendon hydration state that is associated with pain when quantified in specific tissue compartments without the need of invasive tendon biopsies, is of great value for future research focusing on clinical outcome after therapeutic interventions.^36^

The mechanism behind the ultrastructural changes that resulted in a T_2_^*^ decrease in the degenerative tissue of the patellar tendon remains unclear. Theoretically, the decrease of long T_2_^*^ components could result from changes in the macromolecular binding state of water, due to an increase or change of the proteoglycan and glycosaminoglycan content within the degenerative tissue.^37^ Another possible explanation is that within the degenerative tissue, the voxels contained an increasing proportion of (ultra)short T_2_^*^ components over time, for example, due to newly formed collagen fibers as a response to exercise therapy.

A strength of this study is that we performed a large clinical trial that implemented longitudinal UTE‐MRI acquisitions to evaluate T_2_^*^ changes in patients with PT. Other strengths are the advanced methods for image preparation and postprocessing of the UTE‐images, including in‐house developed linear and nonlinear registration tools for a spatial one‐to‐one mapping of voxels that were constant over visits from different UTE‐acquisitions and the analysis of T_2_^*^ in specific tissue compartments of the patellar tendon. We believe that these methods strongly facilitated the analysis of longitudinal data. After performing sensitivity analyses, our findings were consistent with those from the primary analysis and would lead to similar conclusions about the association between decreased T_2_^*^ in the degenerative tissue of the patellar tendon and improved clinical outcome.

## Limitations

We proposed a method to quantify T_2_^*^ in different tissue compartments of the patellar tendon, represented by thresholding the percentage of short T_2_^*^ components voxel‐wisely into 0%–30%, 30%–60%, and 60%–100% short T_2_^*^ components. Despite the observation that these subcategories of voxels with similar T_2_^*^ characteristics visually corresponded well to degenerative tissue, interface and aligned collagen, respectively, there is uncertainty if these subcategories really represent these tissues. This primarily affects the construct validity of this method of T_2_^*^ analysis, and is difficult to overcome without direct histologic confirmation of this subcategorization. The chosen thresholds were based on histogram analysis of the frequency distribution of the percentage short T_2_^*^ components in each voxel, which categorized the T_2_^*^ data in three groups, irrespective of what the absolute values were for the short and long components within each voxel.^19^ Second, the reliability of the T_2_^*^ quantification is most likely insufficient to detect a clinically relevant difference. From previously published data, we know that the coefficient of repeatability was estimated at 2 msec; however, the change in T_2_^*^ after 24 weeks in all athletes was only 1.3 msec (0.6–2.0).^19^ Third, the long acquisition time of our current protocol impedes application in clinical practice. The protocol as implemented was designed to provide an integral sampling of echo times and to provide a high spatial resolution. For this project, this scanning protocol was acceptable, because we did not intend to develop an image protocol that could be used in clinical imaging. For clinical applications, we recommend an abbreviated protocol, for example, as implemented by Fukuda et al.^32^ With only six echo times in the short TE range, they managed to observe a decreased T_2_^*^ in a remodeling anterior cruciate ligament graft 6 months post‐surgery. Forth, a change in the MR acquisition protocol during the trial was required to optimize SNR for better fitting of T_2_^*^ data, which unfortunately lead to exclusion of 11 patients in the final analyses. Moreover, the unbalanced loss of follow‐up between the exercise groups (one in the PTLE group and seven in the EET group), could suggest that the eccentric exercises were less easy to sustain, which seems logical due to the fact that these exercises should be performed with pain.^38^ This may also be reflected by the previously reported lower subjective patient satisfaction reported in the control group.^26^ Finally, despite the fact that the significant decrease in T_2_^*^ after 24 weeks was associated with clinical improvement in our study participants, it is important to note that the validity of the T_2_^*^ parameters in tendinopathy (with tissue histopathology as reference standard) is currently unknown.

## Conclusion

Tissue‐specific T_2_^*^ relaxation times identified with 3D‐UTE‐MRI are associated with symptom severity in athletes with patellar tendinopathy. Decreasing T_2_^*^ relaxation times in the degenerative tissue of the patellar tendon are associated with improved clinical outcome after exercise therapy for patellar tendinopathy, while it is unsuitable as a single predictive measurement at baseline for clinical outcome. While the change in T_2_^*^ was small and the technique has limitations to use in clinical practice, our findings indicate that the hydration state of the patellar tendon is able to change in response to conservative exercise therapy.
